# Effects of Acute Restraint Stress, Prolonged Captivity Stress and Transdermal Corticosterone Application on Immunocompetence and Plasma Levels of Corticosterone on the Cururu Toad (*Rhinella icterica*)

**DOI:** 10.1371/journal.pone.0121005

**Published:** 2015-04-01

**Authors:** Vania Regina de Assis, Stefanny Christie Monteiro Titon, Adriana Maria Giorgi Barsotti, Braz Titon Jr., Fernando Ribeiro Gomes

**Affiliations:** Departamento de Fisiologia, Instituto de Biociências, Universidade de São Paulo, Rua do Matão, trav. 14, 101, 05508-900, São Paulo, SP, Brazil; University of Rouen, France, FRANCE

## Abstract

Glucocorticoid steroids modulate immunocompetence in complex ways with both immunoenhancing and immunosuppressive effects in vertebrates exposed to different stressors. Such bimodal effects have been associated with variation in duration and intensity of the stress response. Given that natural populations have been exposed to a multitude of stressors, a better understanding of the functional association between duration and intensity of the stress response, the resulting changes in glucocorticoid plasma levels and their impact on different aspects of immunocompetence emerges as a cornerstone for vertebrate conservation strategies. We investigated the effects of a restraint challenge (with and without movement restriction), long-term captivity, and transdermal corticosterone application on plasma levels of corticosterone (hereinafter referred to as CORT) and different parameters of innate immunocompetence in the male cururu toads (*Rhinella icterica*). We show that for *R*. *icterica* restraint for 24h proved to be a stressful condition, increasing CORT by 3-fold without consistent immunological changes. However, the application of a more intense stressor (restraint with movement restriction), for the same period, potentiated this response resulting in a 9-fold increase in CORT, associated with increase Neutrophil/Lymphocyte ratio (N:L) and a lower bacterial killing ability (BKA). Transdermal application of corticosterone efficiently mimics repeated acute stress response events, without changing the immune parameters even after 13 days of treatment. Interestingly, long-term captivity did not mitigate the stress response, since the toads maintained 3-fold increased CORT even after 3 months under these conditions. Moreover, long-term captivity in the same condition increased total leukocyte count (TLC) and generated an even greater decrease in BKA, suggesting that consequences of the stress response can be aggravated by time in captivity.

## Introduction

Glucocorticoid hormones are produced by adrenal or interrenal glands, and their release is modulated by several stressors through the activation of the hypothalamic pituitary-interrenal axis (HPI) inectotherms vertebrates [1]. In a short-term period, the activation of the HPI axis may have beneficial effects, such as temporary suppression of reproduction, increased foraging activity, gluconeogenesis, and regulation of the immune response [2,3]. However, when the HPI axis is activated for longer periods, negative functional consequences such as chronic suppression of growth, reproductive and immune function, and neuronal death have been reported [3–5]. Regarding the immune modulation, the immunosuppressive effects of glucocorticoids have been more commonly observed in contexts of intense and chronic activation of the HPI axis. These immunosuppressive effects include inhibition of the synthesis, release and efficiency of several cytokines and other mediators that promote the immune response and inflammatory reactions, and atrophy of lymphoid tissues, particularly the thymus [1,6]. Moreover, immune-enhancing effects have been very commonly observed in the context of low-intensity and short-term activation of the HPI axis, include an increased expression of receptors for different cytokines [6], and redistribution of immune cells within the body, with corresponding increased traffic of leukocytes and enhanced immune function in organs such as the skin [7,8]. Studies have also identified mechanisms involving dendritic cell, neutrophil, macrophage, and lymphocyte trafficking, maturation, and function through which acute stressors may enhance innate as well as adaptive immunity [7,8]. This bimodal effect of glucocorticoids on the immune response can be mediated by different concentrations of these hormones and possibly different receptors [6,9–11]. As an example, glucorticoid increase T-cell response at low concentration, an effect possibly mediated through mineralocorticoid receptors. Otherwise, these hormones decrease the T-cell response at high concentration, an effect possibly mediated through glucocorticoid receptors [9–11].

Experiments conducted in captivity, where conditions can be carefully controlled, are useful for examining complex biological phenomena, such as the inter-relationships between the levels of glucocorticoids and immune function in different stages of the life cycle [12,13]. However, few studies have examined how the captivity itself affects the activation of the HPI axis and immune function. Captive birds (*Calidris canutus*) show reduced antimicrobial killing ability *in vitro* and the number of circulating heterophils and eosinophils when compared to free-ranging individuals [14]. Moreover, the effects of captivity maintenance may change through time, and such changes are presently difficult to predict for different vertebrates [15,16]. Newly captured animals might show an initial strong release of glucocorticoids and a consequent immunosuppression [17]. However, they might habituate to captivity conditions through time, decreasing glucocorticoid plasma levels and improving immune response. Alternatively, the stress response could increase with time, resulting in an even stronger immunosuppression [18–24]. These responses might be species-specific, and might reflect ecological associations through evolutionary history and show important implications for strategies of conservation.

Amphibian populations are undergoing large declines, and the main causes of this decline include habitat loss and fragmentation, occurrence of infectious diseases, habitat pollution, and introduction of exotic species that may become predators/competitors to native species [25,26]. Several infectious agents have been associated with population declines of amphibians including: 1) Chytrid fungus parasite (Bd: *Batrachochytrium dendrobatidis*) [27,28]; 2) Pathogenic bacteria, such as *Aeromonas hydrophilla* [29,30]; and 3) Ranavirus [31]. An interesting fact is that in most cases of mortality events caused by these infectious agents, there were reports of other non-affected amphibian species occupying the same habitat [25]. Amphibians are a vertebrate group that is particularly sensitive to different environmental changes and, consequently, studies of the effects of stressors on immune and reproductive functions are needed to define conservation strategies, including captive breeding [32] and immunization [33].

We investigated the effects of short-term restraint (with and without movement restriction), long-term captivity, and transdermal corticosterone application on plasma levels of corticosterone (hereinafter referred as CORT) and innate immunocompetence in male cururu toads (*Rhinella icterica*). We tested the following hypotheses: 1) Restraint challenge in newly captured toads (maintaining the toads for 24h of captivity) can be considered a stressor, promoting elevation of CORT and reduced immunocompetence relative to baseline values in natural environment; 2) Restraint challenge with movement restriction in newly captured toads (maintained in moistened cloth bags for 24h) can be considered a stronger stressor, promoting more intense effects of CORT elevation and immunossupression than captivity maintenance; 3) Keeping animals in prolonged captivity conditions (three months) could mitigate the effects of restraint challenge for 24h (with or wihout movement restriction); 4) Transdermal corticosterone application should cause an increase in CORT and a reduction in immunocompetence, mimicking the effects of a potent stressor, such as restraint with movement restriction.

## Materials and Methods

### Animals and study site

*Rhinella icterica* is a large toad from the *R*. *marina* group [34]. This species shows geographic distribution associated with forested habitats (Atlantic rain forest), although these toads are common in anthropomorphized areas [34]. Males were collected in February 2012 (*N* = 23), in São Luiz do Paraitinga (23°13′23" S; 45°18′38" WO), São Paulo, Brazil. These individuals were used to test the effects of prolonged captivity stress and for the experimental manipulation of corticosterone levels (transdermal corticosterone application). Twenty additional male toads were collected in January 2013, at the same location, and were used to test the effects of restraint stress with and without movement restriction. Although all these toads were kept during the reproductive season, they were not calling. The collections were performed under authorization from Instituto Brasileiro do Meio Ambiente e dos Recursos Naturais Renováveis (IBAMA, process 17895–1) and laboratory procedures were performed under the approval of the Comissão de Ética no Uso de Animais (CEUA) do Instituto de Biociências da Universidade de São Paulo (CEUA—n° 142/2011).

### Collecting and processing of blood samples

Animals were located by visual inspection, captured and blood was collected in the field (about 200 μl) via cardiac puncture with 1 ml syringes and needles 26Gx1/2'' previously heparinized. The blood samples were considered only if collection was performed within 3 min after animal capture, in order to avoid any influence of the stress of capture and manipulation on hormone levels [35].

All blood samples were identified and kept on ice until they were divided into two aliquots on the same night. One of these aliquots was used for total leukocyte count, blood smear (for further analysis of leukocyte profile), and analysis of hematocrit. The other aliquot was centrifuged to isolate the plasma (4 min at 3000 rpm). Plasma samples (a range 100–150 μl) were stored in cryogenic tubes, and kept in liquid nitrogen until they could be transferred to a -80°C freezer, for posterior hormone assays and analyses of bacterial killing ability.

### Analysis of blood parameters

#### Total leukocyte count (TLC)

On the same night of blood sampling, 5 μl of blood were diluted in 120 μl of saline solution of toluidine blue (0.01%). The toluidine blue stains cells, facilitating differentiation of leukocytes and erythrocytes. Ten microliters of this dilution was placed on a hemocytometer, and TLC were performed under a light microscope (40X objective—Nikon E200, 104c). The number of leukocytes was counted in one quadrant and multiplied by the dilution factor (25X).

#### Hematocrit (HEM)

HEM was calculated as the proportion of blood cells in relation to the total volume of blood after centrifugation of the blood contained in a microhematocrit tube (4 min at 3000 rpm).

#### Leukocyte profile

A drop of blood (about 2 μl) was used to perform each blood smear slide. Two slides were made for each animal and, subsequently, one of these slides was stained with Giemsa solution (10%) and observed under an optical microscope (100X objective, using oil immersion—Nikon E200, 104c). For differential leukocyte counts, 100 leukocytes were counted on each slide, and classified based on morphology as neutrophils, lymphocytes, eosinophils, basophils, and monocytes [36]. Based on the leukocyte profile, the ratio between neutrophils and lymphocytes (N:L) was calculated.

#### Bacterial killing ability (BKA)

This assay was conducted according to [37]. Briefly, plasma samples diluted (1: 20) in Ringer's solution (10 μl plasma: 190 μl Ringer) were mixed with 10 μl of *E*. *coli* working solution (~10^4^ microorganisms). Positive controls consisted of 10 μl of *E*. *coli* working solution in 200 μl of Ringer's solution, and negative control contained 210 μl of Ringer's solution. All samples and controls were incubated for 30 min at 37°C. After the incubation period, 500 μl of tryptic soy broth (TSB) were added to each sample. The bacterial suspensions were thoroughly mixed and 300 μl of each one were transferred (in duplicates) to a 96 wells microplate. The microplate was incubated at 37°C for 2 hours, and thereafter the optical density of the samples was measured hourly in a plate spectrophotometer (wavelength 600 nm), totaling 4 readings. The BKA was calculated according to the formula: *1 - (optical density of sample / optical density of positive control)*, which represents the proportion of killed microorganisms in the samples compared to the positive control. The bacterial killing ability was evaluated at the beginning of the bacterial exponential growth phase.

#### Hormonal assay

*Plasma extraction*: Plasma samples were extracted with ether according to [38]. Briefly, 3 ml of ether was added to 10 μl of each sample, and then vortexed for 30 seconds and centrifuged (4°C, 9 min, at 1800 rpm). Next, the samples were allowed to decant in -80°C freezer for 7 min and the liquid phase was transferred to another tube. These tubes were kept in laminar flow hood at room temperature (20 ± 2°C), until all of the ether had evaporated (approximatelly 24h). The samples were resuspended in EIA buffer and CORT was assayed using EIA kits (number 500655, Cayman Chemical), according to the manufacturer’s instructions.

*Validation of the enzyme-immunoassay*: To validate the use of the Cayman kit for anurans, we tested if the kits were sensitive to detect alterations in CORT in response to: 1) 24h of captivity; and 2) Transdermal corticosterone application in *R*. *icterica*.

Based on previous studies, mean baseline CORT values measured by radioimmunoassay for *R*. *icterica* were 18.5 ng/ml [22]. Given that the standard curve of this Cayman kit is expressed in pg/ml, we knew that large sample dilutions would be necessary. In this way, all samples were run in duplicates, and at three different dilutions. For baseline values, we used 1:50, 1:75 and 1:150 dilutions and after aforementioned stress procedures, we used 1:100, 1:200 and 1:300.

Baseline CORT values for *R*. *icterica* (7.7 ± 6.1 ng/ml) were within the range expected based on [22]. The somewhat lower values measured with EIA reflect the fact that these toads were not calling. Maintenance of *R*. *icterica* in captivity for 24h promoted a 3-fold increase in CORT (20.3 ± 9.5 ng/ml), and transdermal corticosterone application increased CORT in 12-fold (156.9 ± 90.2 ng/ml).

By testing 15 duplicates on each plate, we estimated intra-assay variation to be 8.3% and inter-assay variation was estimated using the average of four intermediate values from the standard curve (as recommended by the kit instructions) and it was 14.8% for *R*. *icterica*. Sensitivity of the assay was 30 pg/ml.

### Phytohemagglutinin (PHA) skin swelling assay

An immunological challenge with PHA was performed to assess the cell-mediated innate immunity, and this procedure was performed 24h after the end of treatment with transdermal corticosterone application.

**Fig 1:**
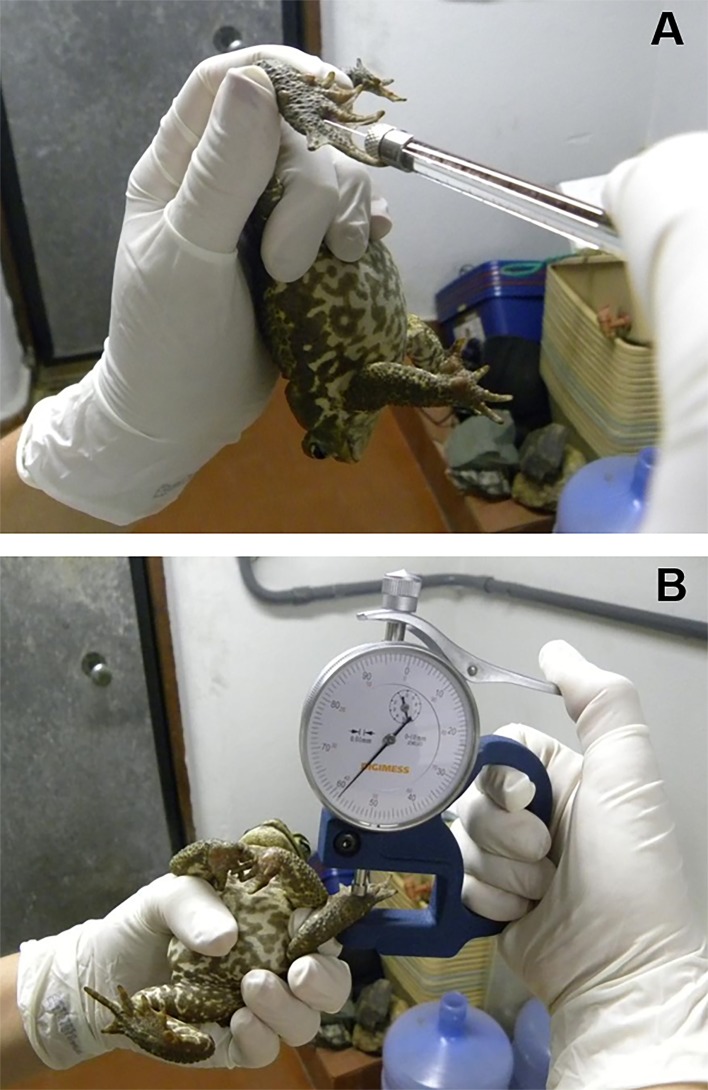
Phytohemagglutinin (PHA) skin-swelling assay. The hind fleshy base of the foot was injected with 10 μl of PHA or sterile saline solution, using a glass syringe (A). The thickness of each hind fleshy base of the foot was measured prior and after 12h of the injection using a thickness gauge: Digimess—0.01mm precision (B) (Photographs by Stefanny C. M. Titon).

The hind fleshy base of the right foot was injected with 10 μl of a 20 mg/ml solution of PHA (Sigma L8754) in sterile saline solution using a 10 μl glass syringes and 30Gx1/2'' needles. As a control, the hind fleshy base of the left foot was injected with 10 μl of sterile saline solution (Fig. 1A) [39–41]. The thickness of each injected hind fleshy base of the foot was measured prior to injection and 12h after injection using a thickness gauge (Digimess—0.01mm precision; Fig. 1B). The swelling in response to PHA was calculated from the proportional increase in hind fleshy base of the foot thickness before and postinjection. Thickness of the fleshy base of the foot was consecutively measured three times and a mean of these values was used for the calculations.

### Experimental manipulation of hormone levels—transdermal corticosterone application

#### Captive maintenance conditions

Immediately after blood collection in the field, animals were placed into individual plastic containers [20L—43.0 (L) x 28.5 (W) x 26.5 (H) cm] with free access to water. The lids of the containers had holes to allow air circulation. Animals were exposed to a natural light cycle and temperatures compatible with a natural thermal regime, until they were transferred to the laboratory. In the laboratory, the animals were kept individually for three months in these containers, with free access to water and objects to provide hiding. Lighting conditions and temperature were kept constant (LD 13:11h—13h of light [light turned on 06h 30min] and 11h of dark [light turned off 19h 30min] and 22 ± 2°C). While in captivity, all animals had free access to water and containers were cleaned and toads fed with crickets and cockroaches once per week.

#### Corticosterone working solution

Based on post-restriction CORT for *R*. *icterica* [22] and on corticosterone concentrations and volumes used by [42] and [43], we have defined our working concentration as 3 μg corticosterone/ 1 μl sesame oil. We applied one 5 μl drop the solution, comprising 15 μg corticosterone per application. The working solution was prepared by diluting 7.5 mg of crystalline corticosterone (Sigma—27840) in 750 μl absolute ethanol. This corticosterone dilution was added to 2.5 ml of sesame oil, and then this mixture was vortexed, and remained in an open vial overnight for ethanol evaporation. All animals received the same amount of hormone application, since no relationship was found between body mass and CORT in the field (r = -0.042; *P* = 0.865). The two aforementioned studies by [42] and [43] found no relationship between hormone levels and body mass or body index and neither study corrected the volumes applied to body mass.

#### Transdermal application

One week before starting the application, a blood sample was collected from each individual (around 200 μl) between 18h and 20h, within 3 min, to assess the immunocompetence and CORT after long-term maintenance in captivity (3 months). Therefore, the animals were divided into three groups: Control (*N* = 7), Placebo (*N* = 8) and Experimental (*N* = 8). Animals from the control group did not receive anything on the skin, but the plastic container was opened and a micropipette with an empty tip was approached to their back (between the front legs), simulating an application. For placebo group, the container was opened and one drop of 5 μl sesame oil was administered on the back of the animals using a micropipette. The same procedure was used for individuals from the experimental group using one drop of 5 μl working solution (a mixture of sesame oil + hormone). The time chosen for the application was between 17h and 19h, with 5 min intervals between individuals, in order to temporally match the baseline samples carried out in the field. Additionally, given that these animals are nocturnal, sampling occurred at a time near to the expected peak of CORT, associated with the onset of the activity period [44].

Water was removed to ensure complete absorption of application for all groups before daily application of treatment and was returned 3h after treatment. The transdermal application treatment occurred for 13 consecutive days and, at the end of this period, a new blood sample was collected from each individual (around 200 μl) between 18h and 20h, 1h after the last application, to assess immune parameters and CORT.

One day after the end of the experiment of transdermal application, animals were tested for subcutaneous inflamation response to PHA. The swelling was measured 12h postinjection of saline and PHA. At the end of this procedure, individuals were euthanatized with an intraperitoneal injection (75mg/kg) of sodium thiopental (Thiopenthax) solution (25mg/ml).

### Comparison between baseline values of CORT and immunocompetence with values after restraint challenge with and without movement restriction

Immediately after blood collection in the field, individuals from the second group of toads captured were randomly placed directly into the individual plastic containers (restraint) or within moistened cloth bags and then in the individual plastic containers (restraint with movement restriction), where they remained for exactly 24h. The lids of the containers had holes to allow air circulation. Animals were exposed to the natural light cycle and temperatures compatible with its natural thermal regime. At the end of 24h, the individuals were bled again to assess measures of immunocompetence and CORT. Upon termination of this experimental protocol, animals were measured (mm), weighed (0.01g) and returned to their collection point at night.

### Statistical analysis

The data were initially analyzed with descriptive statistics and Shapiro-Wilk normality test. Some variables showed absence of normality and were transformed to fit the prerequisites of parametric tests: 1) BKA—arccosine; 2) N:L—ln; and 3) CORT—log_10_. To test for the effects of long-term captivity (three months) on blood parameters (BKA, HEM, TLC, N:L, CORT, neutrophils, lymphocytes, eosinophils, basophils and monocytes) and body mass, we compared these data with those obtained in the field by using paired samples t-test. To test for repeatability in these variables, parametric correlations (Pearson) between data in field and after long-term captivity were used. Correlation tests (Pearson) were also used to investigate possible relationships between variables in the field and after long-term captivity. Treatment effects of transdermal corticosterone application on blood parameters, body mass and response on the PHA skin-swelling challenge were tested by one-way ANOVA with treatment group as a factor. To test for swelling differences between feet that received saline and PHA, we used paired samples t-tests. To compare the variables in the field and after 24h of restraint with and without movement restriction, we used t-tests for paired and independent samples. All analyzes were performed using SPSS version 17.

## Results

### Effects of long-term captivity and transdermal corticosterone application on CORT and immunocompetence

**Fig 2:**
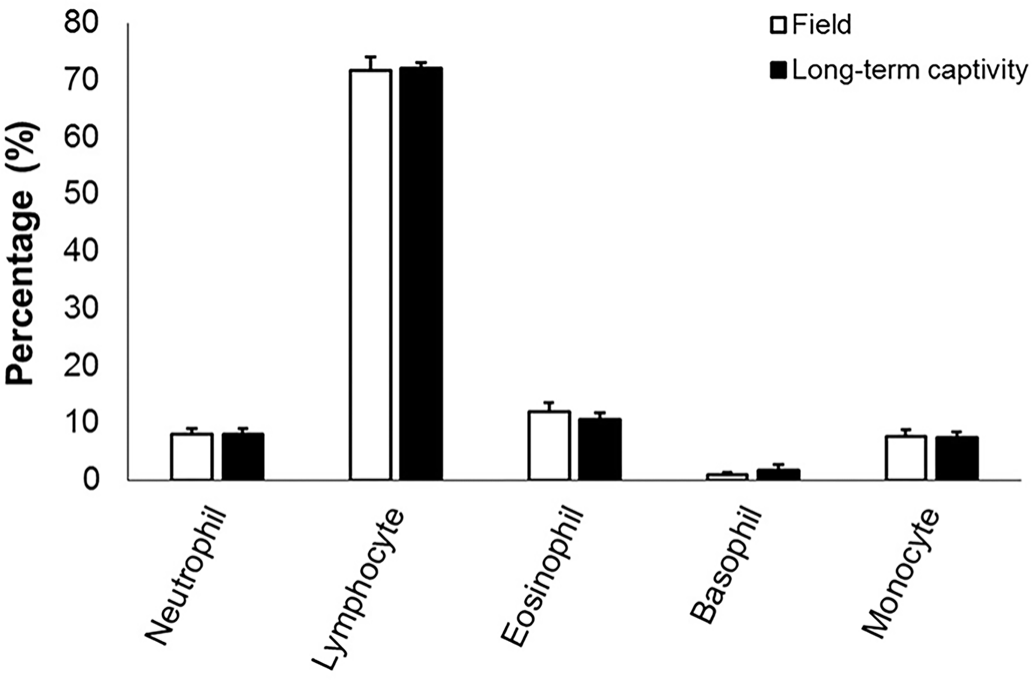
Leukocyte profile of *Rhinella icterica*. Leukocyte profile of adult males of *Rhinella icterica* under field conditions and after long-term captivity (three months). Bars represent mean ± standard error. *N* is the same for all variables (*N* = 23).

Descriptive statistics for males of *R*. *icterica* in field conditions, after long-term captivity (3 months), and at the end of the transdermal corticosterone application are shown in Table 1. Leukocyte profile for these males, in the field and after long-term captivity, is shown in Fig. 2.

**Table 1:** Descriptive statistics of blood parameters and body mass for individuals of *R*. *icterica* under field conditions, after long-term captivity (three months), and at the end of the experiment of transdermal corticosterone application for 13 days.

			Parameter	N	Minimum	Maximum	Mean ± SD
**FIELD**			BKA (%)	22	0.00	100.00	67.45 ± 40.65
			HEM (%)	22	7.00	45.00	29.32 ± 11.46
			TLC (cells/μl)	21	450	2900	1285 ± 715
			N:L	23	0.00	0.36	0.13 ± 0.09
			CORT (ng/ml)	19	5.56	42.64	12.85 ± 8.38
		Leukocyte Profile (%)	Neutrophil	23	0.00	20.00	8.09 ± 4.69
			Lymphocyte	23	49.00	97.00	71.60 ± 11.68
			Eosinophil	23	0.00	37.00	11.96 ± 8.16
			Basophil	23	0.00	4.00	1.04 ± 1.22
			Monocyte	23	1.00	20.00	7.74 ± 5.15
			Body mass	23	70.99	195.20	127.95 ± 34.74
**LONG-TERM CAPTIVITY**			BKA (%)	23	0.00	84.00	40.17 ± 28.30
			HEM (%)	23	6.00	25.00	16.02 ± 4.80
			TLC (cells/μl)	23	675	5075	2510 ± 1260
			N:L	23	0.01	0.41	0.12 ± 0.10
			CORT (ng/ml)	23	7.48	93.84	39.90 ± 23.89
		Leukocyte Profile (%)	Neutrophil	23	1.00	22.00	8.00 ± 5.20
			Lymphocyte	23	54.00	88.00	72.09 ± 10.84
			Eosinophil	23	1.00	25.00	10.70 ± 7.26
			Basophil	23	0.00	10.00	1.70 ± 2.63
			Monocyte	23	0.00	17.00	7.52 ± 4.64
			Body mass	23	78.10	175.52	122.69 ± 27.84
**TRANSDERMAL CORTICOSTERONE APPLICATION**	***Control***		BKA (%)	7	84.00	97.00	90.57 ± 4.65
			HEM (%)	7	13.00	21.00	18.00 ± 2,88
			TLC (cells/μl)	7	1850	3900	2782 ± 777
			N:L	7	0.02	0.39	0.16 ± 0.14
			CORT (ng/ml)	7	14.34	39.69	29.67 ± 10.32
		Leukocyte Profile (%)	Neutrophil	7	2.00	19.00	9.86 ± 7.99
			Lymphocyte	7	49.00	84.00	69.29 ± 12.05
			Eosinophil	7	2.00	14.00	8.86 ± 4.49
			Basophil	7	0.00	16.00	5.14 ± 5.79
			Monocyte	7	2.00	14.00	6.86 ± 4.41
			Body mass	7	89.82	179.98	130.48 ± 33.59
	***Placebo***		BKA (%)	7	0.00	95.00	56.43 ± 40.57
			HEM (%)	7	7.00	27.00	17.57 ± 6.27
			TLC (cells/μl)	7	600	3925	2439 ± 1313
			N:L	7	0.06	0.66	0.22 ± 0.20
			CORT (ng/ml)	7	7.88	54.43	26.92 ± 16.22
		Leukocyte Profile (%)	Neutrophil	7	5.00	31.00	13.14 ± 8.55
			Lymphocyte	7	47.00	87.00	70.43 ± 12.58
			Eosinophil	7	4.00	21.00	9.57 ± 5.86
			Basophil	7	0.00	7.00	2.71 ± 2.43
			Monocyte	7	1.00	11.00	4.14 ± 3.58
			Body mass	7	76.27	144.95	112.72 ± 21.78
	***Experimental***		BKA (%)	7	0.00	98.00	74.14 ± 33.68
			HEM (%)	7	10.00	27.00	16.43 ± 6.78
			TLC (cells/μl)	7	750	4375	3064 ± 1283
			N:L	7	0.13	0.64	0.25 ± 0.18
			CORT (ng/ml)	7	56.21	324.82	156.96 ± 90.20
		Leukocyte Profile (%)	Neutrophil	7	10.00	25.00	15.43 ± 4.86
			Lymphocyte	7	39.00	80.00	69.43 ± 14.72
			Eosinophil	7	2.00	10.00	6.57 ± 2.99
			Basophil	7	0.00	18.00	3.57 ± 6.60
			Monocyte	7	2.00	10.00	5.00 ± 3.06
			Body mass	7	82.51	173.87	126.40 ± 32.57

**BKA**: Bacterial killing ability; **HEM**: Hematocrit; **N:L**: Neutrophil/ Lymphocyte ratio; **TLC**: Total leukocytes count; **CORT**: Corticosterone plasma levels.

**Fig 3:**
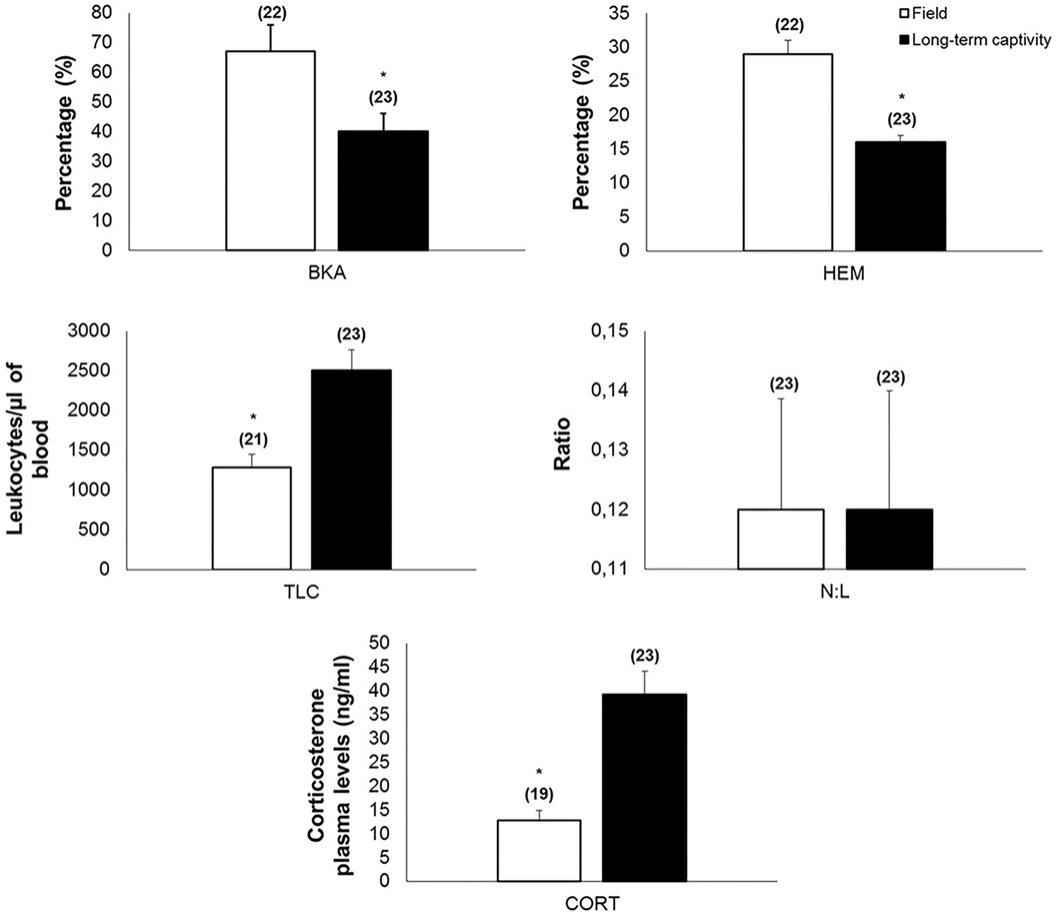
Blood paramenters—comparison between baseline and after long-term captivity. Comparison of blood parameters of toads (*Rhinella icterica*) in the field and after long-term captivity (three months). Bars represent mean ± standard error with *N* in parentheses. **BKA**: Bacterial killing ability; **HEM**: Hematocrit; **N:L**: Neutrophil/ Lymphocyte ratio; **TLC**: Total Leukocytes Count; **CORT**: Corticosterone plasma levels. *P ≤ 0.001.

Long-term captivity resulted in a mean reduction of 41% in BKA and 45% in HEM, in addition to a mean increase of 3-fold in CORT and 2-fold in TLC, without changing the leukocyte profile, N:L and body mass (Fig. 3, Table 2). Individuals with higher HEM also had higher BKA in the field (r = 0.564, *P* = 0.008) and in captivity (r = 0.498, *P* = 0.016), and individuals with higher TLC also showed higher HEM in field (r = 0.539, *P* = 0.014) and in captivity (r = 0.534, *P* = 0.010). Individuals showed consistent variation for BKA (r = 0.448, *P* = 0.037) and body mass (r = 0.754, *P* ≤ 0.001), when data in the field and after long-term captivity were compared.

**Table 2:** Comparison between blood parameters and body mass for individuals of *R*. *icterica* under field conditions and after long-term captivity (three months).

	Parameter	*t-value*	DF	*P*
	BKA (%)	4.220	21	**≤ 0.001**
	HEM (%)	5.230	21	**≤ 0.001**
	TLC (cels/μl)	-4.965	20	**≤ 0.001**
	N:L	0.204	22	0.840
	CORT (ng/ml)	-5.659	18	**≤ 0.001**
Leukocyte Profile (%)	Neutrophil	0.056	22	0.956
	Lymphocyte	-0.139	22	0.891
	Eosinophil	0.695	22	0.494
	Basophil	-1.066	22	0.298
	Monocyte	0.179	22	0.860
	Body mass	1.103	22	0.282

Paired samples T-Test. Tests significant at 0.05 are in bold. **BKA**: Bacterial killing ability; **HEM**: Hematocrit; **N:L**: Neutrophil/ Lymphocyte ratio; **TLC**: Total leukocytes count; **CORT**: Corticosterone plasma levels.

**Fig 4:**
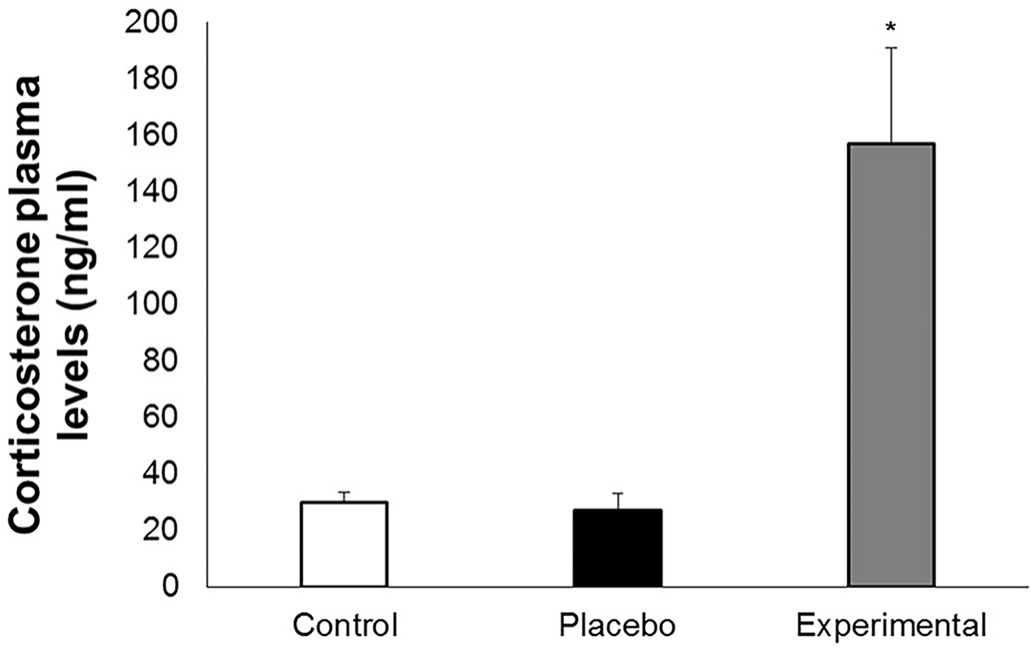
Corticosterone plasma levels after transdermal application. Corticosterone plasma levels at the end of the experiment of transdermal application in *Rhinella icterica*. Bars represent mean ± standard error. *N* is the same for all groups (*N* = 7). *P ≤ 0.010.

Transdermal corticosterone application for 13 days produced a 6-fold average increase in CORT compared to values found for the Placebo and Control groups (F_2,18_ = 20.693, *P* ≤ 0.001, Fig. 4) 1h post-application, without changing any other blood parameter or body mass (*P* ≥ 0.115).

PHA injection caused an increase in hind fleshy base of foot thickness after 12hours (t = -3.201, *P* = 0.004), whereas saline did not (t = 0.747, *P* = 0.464). Transdermal corticosterone application did not alter the response to injections of PHA (F_2,18_ = 0.197, *P* = 0.823) and saline (F_2,18_ = 0.632, *P* = 0.543).

### Effects of restraint challenge with and without movement restriction on CORT and immunocompetence

Descriptive statistics for males of *R*. *icterica* in the field, after 24h of restraint with or without movement restriction are shown in Table 3.

**Table 3:** Descriptive statistics of blood parameters and body mass for individuals of *R*. *icterica* under field conditions and after restraint challenge with and without movement restriction.

			Parameter	N	Minimum	Maximum	Mean ± SD
**FIELD**			BKA (%)	20	52.00	100.00	94.00 ± 12.00
			HEM (%)	20	11.00	55.00	33.00 ± 10.00
			TLC (cells/μl)	20	750	5550	2998 ± 1322
			N:L	20	0.08	0.63	0.21 ± 0.15
			CORT (ng/ml)	20	0.76	23.80	7.71 ± 6.12
		Leukocyte Profile (%)	Neutrophil	20	6.00	31.00	13.30 ± 6.34
			Lymphocyte	20	35.00	81.00	69.10 ± 11.10
			Eosinophil	20	4.00	33.00	12.40 ± 7.16
			Basophil	20	0.00	10.00	1.35 ± 2.28
			Monocyte	20	0.00	11.00	3.90 ± 3.00
**AFTER 24H OF MAINTENANCE IN CAPTIVITY**	***Restraint without movement restriction***		BKA (%)	10	0.00	100.00	82.00 ± 32.00
			HEM (%)	10	11.00	45.00	29.00 ± 12.00
			TLC (cells/μl)	10	700	3400	1903 ± 893
			N:L	10	0.12	0.65	0.33 ± 0.18
			CORT (ng/ml)	9	4.59	36.50	20.32 ± 9.48
		Leukocyte Profile (%)	Neutrophil	10	9.00	32.00	18.70 ± 8.03
			Lymphocyte	10	44.00	78.00	62.10 ± 11.06
			Eosinophil	10	4.00	32.00	12.90 ± 7.58
			Basophil	10	0.00	3.00	0.70 ± 1.16
			Monocyte	10	0.00	15.00	5.60 ± 5.17
	***Restraint with movement restriction***		BKA (%)	10	45.00	100.00	87.00 ± 19.00
			HEM (%)	10	9.00	38.00	30.00 ± 8.00
			TLC (cells/μl)	10	1300	3575	2323 ± 665
			N:L	10	0.12	1.81	0.66 ± 0.53
			CORT (ng/ml)	10	7.39	165.22	66.40 ± 48.85
		Leukocyte Profile (%)	Neutrophil	10	10.00	56.00	28.80 ± 14.62
			Lymphocyte	10	31.00	82.00	55.10 ± 17.48
			Eosinophil	10	1.00	24.00	9.80 ± 7.67
			Basophil	10	0.00	5.00	1.50 ± 1.90
			Monocyte	10	1.00	8.00	4.80 ± 2.44
			Body mass	20	44.20	188.60	119.88 ± 38.66

**BKA**: Bacterial killing ability; **HEM**: Hematocrit; **N:L**: Neutrophil/ Lymphocyte ratio; **TLC**: Total leukocytes count; **CORT**: Corticosterone plasma levels.

**Fig 5:**
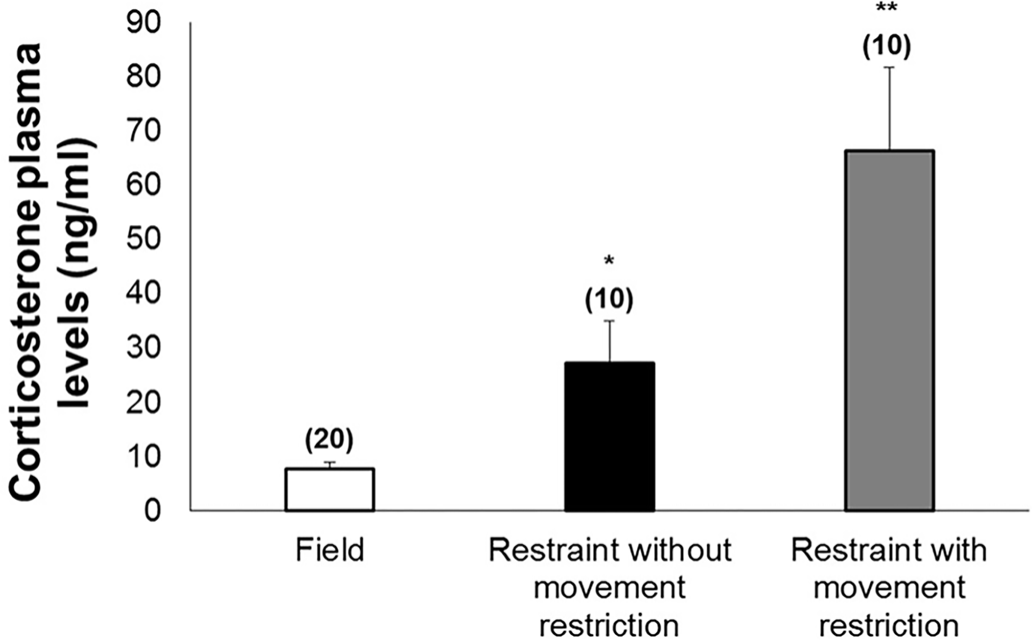
Baseline and after restraint corticosterone plasma levels. Corticosterone plasma levels of toads (*Rhinella icterica*) under field conditions and after 24h of restraint challenge with and without movement restriction. Bars represent mean ± standard error with *N* in parentheses. *P ≤ 0.012; **P ≤ 0.001.

Individuals kept in captivity within plastic containers for 24h (restraint without movement restriction) showed a mean decrease of 38% in TLC and 3-fold increase in CORT (Fig. 5), without changes in other measured parameters (Table 4). Otherwise, individuals maintained within moistened cloth bags for 24h (restraint with movement restriction), showed a mean decrease of 12% in BKA, a 4-fold increase in N:L ratio, and a 9-fold increase in CORT (Fig. 5), without changes in other measured parameters (Table 4).

**Table 4:** Comparison between blood parameters for individuals of *R*. *icterica* under field conditions and after restraint challenge with and without movement restriction.

			Parameter	*t-value*	DF	*P*
**AFTER 24H OF MAINTENANCE IN CAPTIVITY**	***Restraint without mevement restriction***		BKA (%)	0.916	9	0.383
			HEM (%)	0.417	9	0.687
			TLC (cells/μl)	2.352	9	**0.043**
			CORT (ng/ml)	-3.352	9	**0.012**
			N:L	-0.753	9	0.471
		Leukocyte Profile (%)	Neutrophil	-0.883	9	0.400
			Lymphocyte	0.931	9	0.376
			Eosinophil	-0.589	9	0.570
			Basophil	0.228	9	0.825
			Monocyte	-0.686	9	0.510
	***Resraint with movement restriction***		BKA (%)	2.059	9	0.035*
			HEM (%)	1.310	9	0.223
			TLC (cells/μl)	1.679	9	0.127
			CORT (ng/ml)	-5.875	9	**≤ 0.001**
			N:L	-3.074	9	**0.013**
		Leukocyte Profile (%)	Neutrophil	-4.281	9	**0.002**
			Lymphocyte	3.396	9	**0.008**
			Eosinophil	1.667	9	0.130
			Basophil	0.415	9	0.688
			Monocyte	-1.385	9	0.200

Paired samples T-Test. Tests significant at 0.05 are in bold.

*Value corresponding to one-tailed test.

**BKA**: Bacterial killing ability; **HEM**: Hematocrit; **N:L**: Neutrophil/ Lymphocyte ratio; **TLC**: Total leukocytes count; **CORT**: Corticosterone plasma levels.

When the groups maintained in captivity for 24h in different conditions (restraint with x without movement restriction) were compared, we found 2.4-fold higher CORT and a 2-fold higher N:L ratio for the toads restrained with movement restriction, without changing any other blood parameters (Table 5).

**Table 5:** Comparison between blood parameters and body mass for individuals of *R*. *icterica* after 24h of restraint challenge with and without movement restriction.

	Parameter	*t-value*	DF	*P*
	BKA (%)	-0.266	18	0.793
	HEM (%)	-0.332	18	0.743
	TLC (cels/μl)	-1.193	18	0.248
	CORT (ng/ml)	-2.093	18	**0.051**
	N:L	-1.879	18	0.038*
Leukocyte Profile (%)	Neutrophil	-1.637	18	0.119
	Lymphocyte	1.070	18	0.299
	Eosinophil	1.321	18	0.203
	Basophil	-0.994	18	0.333
	Monocyte	0.443	18	0.663
	Body mass	0.497	18	0.625

Paired samples T-Test. Tests significant at 0.05 are in bold.

*Value corresponding to one-tailed test.

**BKA**: Bacterial killing ability; **HEM**: Hematocrit; **N:L**: Neutrophil/ Lymphocyte ratio; **TLC**: Total leukocytes count; **CORT**: Corticosterone plasma levels.

## Discussion

### Effects restraint challenge and long-term captivity on CORT and immunocompetence

As previously observed for other tetrapods, including anurans kept under captivity for hours to days [21–23,45–50], captivity maintenance for 24h increased CORT in *R*. *icterica* when compared to values found for free individuals in the field. These data support the interpretation that captivity is a stressor for these animals. Moreover, such increase in CORT levels associated with captivity is dependent on the restriction level imposed. While restraining toads within large plastic containers promoted a 3-fold increase in CORT, restraining toads within moistened bags, for the same period, increased CORT by nine times the free-range values. Previously similar results for *Rhinella* were shown in [22,23], but in both studies, blood samples after the movement restriction have been collected during the day, while the basal samples were collected during the night. This difference in time of blood sampling employed by previous studies restritcted their conclusions about the scope of stress-response. Interestingly, the group of toads maintained for three months in captivity also showed a 3-fold increase in CORT when compared to the free-range values, suggesting that captivity remains a stressor for a long time, without reduction of the stress response.

Regarding the immune parameters, we found an increase in N:L in toads restrained with movement restriction when compared to both field conditions and restraint without movement restriction. It is known that the increase in CORT promotes changes in transmigration patterns of different leukocyte types between blood and other tissues. This modulation commonly results in a reduction in circulating lymphocyte levels and an increase in production and influx of neutrophils into the blood stream, consequently generating an increase in N:L ratio [51–54]. Although movement restriction increased CORT and N:L in *R*. *icterica*, as previously observed for other tetrapods [54–58], a direct correlation between these variable has not been found. Given that the toads exposed to restraint without movement restriction showed increased CORT without changes in N:L, our results suggest that only stronger stressors, associated with higher CORT, are associated with increased N:L.

Additionally, we have observed somewhat contradictory changes in TLC associated with short-term and long-term captivity stress. While the restraint without movement restriction of toads by 24h decreased TLC by 38%, three months in captivity increased TLC by 2-fold. The initial decrease in TLC might be associated to the short time interval between blood samples collected. However, the same sampling interval was applied to the restraint with movement restriction group, and such effect was not observed for these toads. Increased TLC has been used as a measure of stress (for example, [59]) and injections of steroid hormones in horses promoted doubling TLC within 2h [60]. In this way, the increased CORT along with higher TLC support the interpretation that long-term captivity represents a stressor for these toads.

Restraint with movement restriction and long-term captivity promoted 12% and 41% reduction respectively in the ability to eliminate *E*. *coli* in *R*. *icterica*, when compared to field conditions. Reduced BKA had been previously observed in response to restraint challenge in *R*.*marina* [23], and due to the captivity maintenance in birds [14]. Thus, our data demonstrate that the stress associated with restraint and long-term captivity results in immunosuppression, at least in the humoral innate response [12–14,23,61]. Moreover, these results also corroborate the hypothesis that the stronger acute stressor, associated with higher CORT, promote more intense immunosuppression. Long-term captivity maintenance is associated with an even higher reduction in BKA in *R*. *icterica*, reinforcing the point that the stress response of these toads does not reduce the stress level even after three months.

Along with the previously described effects of long-term captivity, toads showed reduced HEM in this condition. Due to their high tolerance of dehydration and low skin resistance to water loss, toads are subject to high rates of water loss by evaporation in the terrestrial environment [62,63]. In this way, the reduction of HEM and BKA after three months in captivity could reflect a higher degree of hydration associated with free access to water under these conditions. However, the lack of long-term captivity effect on body mass and the 2-fold increased TLC do not support the possibility of a pronounced effect of hydration level on these results. Although treatment with hydrocortisone stimulated the production of erythrocytes in frogs [64], the relationship between erythropoiesis and glucocorticoids remains uncertain even in mammals, with evidence of stimulation and inhibition on erythropoiesis depending on dose and time of glucocorticoids application [65,66].

The positive correlations observed between BKA, TLC and HEM in the field and after long-term captivity, along with the evidence of increased stress response and reduced immunocompetence, indicate that these three variables might be used as indexes of allostatic state (definition according to [67]), with possible implications for detection of stressors in natural populations of this species and applications in conservation strategies [68,69]. Moreover, BKA showed repeatability when data from the field and after the long-term captivity were compared. Although long-term captivity reduced mean BKA, individuals characterized by higher BKA in the field continued to show higher values after three months in captivity. Previous studies also demonstrated the maintenance of the mean BKA in males from the same population of *R*. *ornata* collected during vocal activity in two different breeding seasons, and patterns of interspecific variation in BKA consistent with the existence of phylogenetic signal for this variable in anurans [22,37]. Given that the repeatability is a prerequisite for the detection of inter-individual variation, and inter-individual variation represents the substrate for the action of natural selection, these data reinforce the possibility of adaptive interpretations for interspecific variation of BKA in anurans [22,37]. A next step in this direction would be, however, the detection of heritability for this trait [70–72].

### Transdermal corticosterone application

Transdermal corticosterone application resulted in a 6-fold increase in CORT on animals in the experimental group compared to the values from control and placebo groups, and 12-fold when compared to baseline values (obtained in the field). The volume and concentration applied of the hormone was sufficient to increase CORT in these animals, as previously observed for lizards [42] and salamanders [43]. Additionally, the lack of differences in CORT between our control and placebo groups shows the lack of effect of the manipulation performed on hormone levels. Despite the considerable increase in CORT on animals in experimental groups, no other blood parameter showed significant differences between groups at the end of the experiment.

In the study carried out by [43] male salamanders (*Desmognathus ocoee*) were treated for 9 consecutive days, with corticosterone through an application of a dermal patch that lasted 30 min. The authors found significantly elevated CORT one hour after removal of the dermal patch, but no differences were found between treated and non-treated animals 8h after removing the patch. Additionally, this treatment resulted in repeated acute elevations CORT over the 9 days, without changes in baseline values [43]. In our study, given that the samples were always collected 1h post-application, we were not able to explain the temporal dynamics of CORT changes due to treatment. However, in another experiment with males from this same species, collecting blood after 1h, 6h and 12h of application, we confirmed that differences in CORT between experimental and placebo groups occurred only 1h post-application, both for the first and for the last day of a 30 days period of daily application (Assis et al., unpublished results). In this way, these results suggest that the transdermal corticosterone application in *R*. *icterica* resulted in daily transient peaks of CORT, instead of promoting sustained hormonal peaks that would mimic a chronic stress condition [8,43,73]. The absence of changes in the immune parameters, unlike the observed effects of long-term captivity, also corroborates this interpretation.

Another possible explanation for the absence of changes in immune parameters following the transdermal corticosterone application would be that the experiment was conducted for an insufficient time. However, 30 days of treatment also did not change the immune parameters (Assis et al., unpublished results). An experimental protocol involving subcutaneous corticosterone implants would be interesting to tell apart the influence of acute and chronic exposition to corticosterone on immune parameters in these toads. An additional possibility is that the contrast of the effects of long-term captivity and transdermal corticosterone application on the immune variables in this study is the expression of a bimodal effect of this hormone [1,6,9–11,74]. In rats, for example, it is known that corticosterone may potentiate or inhibit the production of melatonin by pineal gland and leukocytes, indirectly modulating the inflammatory process, and this bimodal effect depends on corticosterone concentration [75,76]. Application of different concentrations of corticosterone, followed by melatonin and immunocompetence measures, would be needed to test this hypothesis of melatonin mediated bimodal action of corticosterone in anurans.

Despite the increased thickness of hind fleshy base of foot injected with PHA when compared to the saline control, the treatment of transdermal corticosterone application did not affect the response to PHA. Although a previous study with *R*. *marina* has shown a progression of swelling with the time postinjection of PHA, reaching maximum values after 24h, the cell infiltration associated with innate response occurred 12h postinjection [41]. Moreover, previous results obtained in our laboratory for a tree-frog (*Hypsiboas albopunctatus*), showed maximum swelling-response to PHA at 12h postinjection, and hind fleshy base of feet returned to basal conditions at 24h postinjection (Titon et al., unpublished data). These observations guided our initial choice to standardize the swelling measurements to 12h postinjection. However, we cannot rule out the possibility that an effect of corticosterone treatment would be more pronounced at 24h postinjection for this species. The measures of hind fleshy base of foot thickness at later postinjection intervals in further studies will provide information needed to clarify this issue.

In summary, we showed that for *R*. *icterica*, restraint for 24h was a stressful condition, increasing CORT in 3-fold without consistent immunological changes. However, the application of a more invasive stress protocol (restraint with movement restriction) for the same period potentiated this response, resulting in 9-fold increase in CORT, associated with increased N:L ratio and lower BKA. Transdermal application of corticosterone efficiently mimicked repeated acute stress response events, without changing immune parameters even after 13 days of treatment. Interestingly, long-term captivity did not mitigate the stress response, since these toads maintained 3-fold increased CORT even after 3 months under these conditions. Moreover, long-term captivity increased TLC and generated an even stronger decrease in BKA, suggesting that consequences of the stress response can be aggravated by time in captivity. Such strong immune consequences of response to chronic stress in toads, if generalized to other stressors such as environmental pollution and habitat loss fragmentation, might show important impact on fitness of natural populations. Additionally, other physiological functions crucial to fitness, such as reproduction, might also be disrupted by response to chronic stress. The effects of biologically relevant chronic stressors on immune response and other physiological functions, such as growth and reproduction, represent important avenues of investigation.
